# Chromosome-breakage genomic instability and chromothripsis in breast cancer

**DOI:** 10.1186/1471-2164-15-579

**Published:** 2014-07-09

**Authors:** Ewa Przybytkowski, Elizabeth Lenkiewicz, Michael T Barrett, Kathleen Klein, Sheida Nabavi, Celia MT Greenwood, Mark Basik

**Affiliations:** Department of Oncology, Lady Davis Institute for Medical Research, McGill University, 3755 Cote Ste-Catherine Road, Montreal, Quebec H3T-1E2 Canada; Department of Surgery, McGill University, Montreal, QC Canada; Pharmaceutical Genomics Division, Translational Genomics Research Institute, Scottsdale, Arizona USA; Lady Davis Institute for Medical Research, Jewish General Hospital, Montreal, QC Canada; Beth Israel Deaconess Medical Center & Center for Biomedical Informatics, Harvard Medical School, Boston, Massachusetts USA; Department of Epidemiology, Biostatistics and Occupational Health, McGill University, Montreal, QC Canada

**Keywords:** Breast cancer, Genomic instability, Array CGH, Copy number alterations, Chromosomal breakpoints, Chromothripsis, Gene amplification

## Abstract

**Background:**

Chromosomal breakage followed by faulty DNA repair leads to gene amplifications and deletions in cancers. However, the mere assessment of the extent of genomic changes, amplifications and deletions may reduce the complexity of genomic data observed by array comparative genomic hybridization (array CGH). We present here a novel approach to array CGH data analysis, which focuses on putative breakpoints responsible for rearrangements within the genome.

**Results:**

We performed array comparative genomic hybridization in 29 primary tumors from high risk patients with breast cancer. The specimens were flow sorted according to ploidy to increase tumor cell purity prior to array CGH. We describe the number of chromosomal breaks as well as the patterns of breaks on individual chromosomes in each tumor. There were differences in chromosomal breakage patterns between the 3 clinical subtypes of breast cancers, although the highest density of breaks occurred at chromosome 17 in all subtypes, suggesting a particular proclivity of this chromosome for breaks. We also observed chromothripsis affecting various chromosomes in 41% of high risk breast cancers.

**Conclusions:**

Our results provide a new insight into the genomic complexity of breast cancer. Genomic instability dependent on chromosomal breakage events is not stochastic, targeting some chromosomes clearly more than others. We report a much higher percentage of chromothripsis than described previously in other cancers and this suggests that massive genomic rearrangements occurring in a single catastrophic event may shape many breast cancer genomes.

**Electronic supplementary material:**

The online version of this article (doi:10.1186/1471-2164-15-579) contains supplementary material, which is available to authorized users.

## Background

Breast cancer is a heterogeneous disease showing diverse clinical characteristics and various responses to therapies. Although for clinical purposes, breast cancers are categorized based on the expression of a few key markers, such as the estrogen receptor (ER), the progesterone receptor (PR) and the human epidermal growth factor receptor 2 (HER2) whose levels are assessed using immunohistochemistry (IHC)-based methods [1], recent genomic studies have uncovered much greater heterogeneity in breast cancers. Gene expression profiles in particular have shown good potential to refine breast tumors categories [2, 3]. The most recent large-scale study combining DNA copy number changes with gene expression profiles (METABRIC) led to the identification of 10 novel “integrative” subclasses with a different prognosis according to subclass [4].

Most studies of DNA copy number changes in breast tumors report on the potential clinical value of altered genes within these changes, while few have focused on the breakage events leading to the rearrangements themselves. Indeed a closer examination of array CGH data suggests that commonly studied amplicons such as the one containing the ERBB2 oncogene (encoding for the HER2 receptor) are very heterogeneous, containing breaks not only at the beginning and the end of the amplicons, but also within the amplicons themselves. Genomic instability is usually measured as the proportion of the genome that is altered rather than the number or patterns of those changes. This complexity has not been properly addressed before.

In this study, we used array CGH data to evaluate the number of putative chromosomal breaks underlying gene amplification and deletion events. In order to increase the sensitivity of this approach, we used DNA extracted from tumor nuclei sorted according to ploidy. This approach enabled the removal in many cases of nuclei originating from admixed non-neoplastic cells from the analysis, revealing subtle amplicons and deletions, as well as intra-amplicon and intra-deletion breaks not previously reported. In a relatively small sample size enriched for high-risk breast cancers, we observed frequent breakpoints patterns and configurations of DNA breaks suggestive of chromothripsis, a recently reported phenomenon of genomic catastrophe affecting various chromosomes [5, 6]. Finally, we observed differences in chromosomal breakage patterns between different clinical breast cancer subtypes, We show that a relatively simple approach to the analysis of genomic data (much less detailed than sequencing) can be very informative when used on highly purified tumor biopsies and can provide new insights into the biology of known clinical categories of breast cancer.

## Results

### Identification and flow sorting of subpopulations from tumor specimens

**Figure 1:**
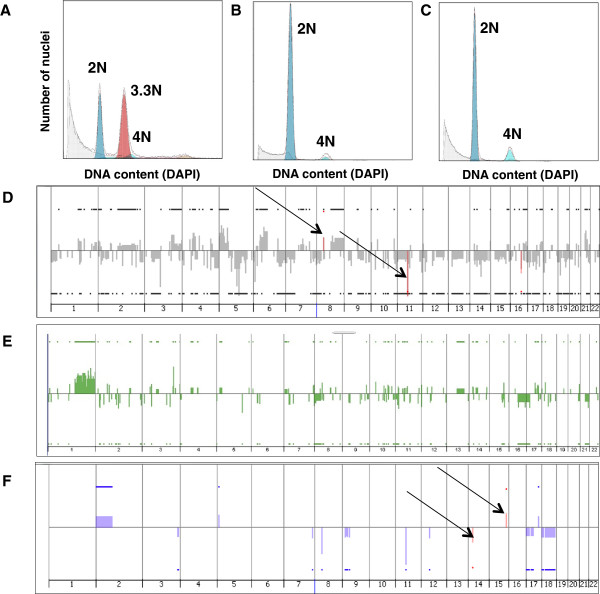
**Flow sorting profiles of tumor specimens and copy number patterns associated with different tumor subpopulations.** Flow sorting profiles **(A-C)** and copy number patterns (genome-wide views) identified with the ADM-2 algorithm **(D-F)**. **A)** Aneuploid profile of tumor T52, **B)** diploid profile of tumor T221, **C)** diploid-like profile of tetraploid tumor T190, **D)** copy number pattern in 3.5 N highly aberrant fraction from aneuploidy tumor T210 (gray) superimposed on pattern found in 2N non-aberrant fraction from the same tumor (red) showing only common CNVs (arrows), **E)** copy number pattern in 2N highly aberrant fraction from diploid tumor T221 shown in green, **F)** copy number pattern in 4N aberrant fraction from tetraploid tumor T190 (blue) superimposed on pattern in 2N non-aberrant fraction from the same tumor (red) showing only common CNVs (arrows).

Primary tumor samples were obtained from high-risk breast cancer patients defined as at least stage 2 or higher breast cancer and/or < 50 years old. Tumor samples typically contain various proportions of tumor cells and normal cells as well as components of stroma and infiltrating lymphocytes. Unlike most tumor cells, these stromal cells do not show variations in DNA content nor DNA copy number alterations [7]. To identify and sort pure diploid and/or aneuploid tumor subpopulations, we measured DNA content in DAPI-labeled nuclei isolated from frozen primary breast tumor specimens [8]. Flow sorting for fractions with different ploidy values was performed on 48 frozen tumor samples of high risk breast tumors. 27 tumor samples (56.3% of the total) showed clear aneuploid cell cycle profiles (Figure 1A). Six of these tumor samples contained inadequate number of nuclei for DNA extraction and were not analyzed further. Thus aneuploid subpopulations sorted from 21 tumors (one subpopulation per tumor) were analyzed with array CGH. All of these DNA samples carried genomic alterations, while 2N subpopulations from these tumors either did not carry any genomic aberrations or carried few aberrations, which localized in all cases to sites of common copy number variations (CNVs) as per the Toronto CNV database. Thus the 2N subpopulations most likely represent contaminating stroma (Figure 1D). 21 tumors out of 48 (43.8%) showed diploid-like profiles (Figure 1B and C). Diploid profiles are more difficult to interpret since diploid sub-populations could represent truly diploid or near-diploid tumor nuclei mixed with nuclei originated from stromal cells, tetraploid tumors (T) with stromal contaminants or normal stroma only. Thirteen (13) of 21 diploid tumors had sufficient numbers of nuclei for DNA extraction and array CGH. Array CGH revealed genomic aberrations in 2N and/or 4N fractions in 8 tumors (Figure 1E and F). Samples with an aberrant 2N fraction were considered as truly diploid or near-diploid tumors (D). The five samples with non-aberrant 2N and 4N fractions were thought to originate from completely non-aberrant tumors or normal stroma and were excluded from further analysis. Overall, in 23 of 29 analyzed samples (21 aneuploid and 2 tetraploid), we are confident about having obtained a highly pure fraction of cancer nuclei. In the other 6 cases (D or D/T), tumor nuclei could be admixed with normal nuclei to a significant extent. Table 1 summarizes all flow sorting results.

**Table 1:** **Summary of flow sorting results**

FACS profile	Sample description	Included in thev study	Excluded from the study
Aneuploid	Sufficient number of aneuploidy nuclei	21	
	Insufficient number of nuclei		6
Diploid	Diploid tumors	2	
	Diploid/tetraploid tumors	6	
	Non-aberrant tumors (or stroma only)		5
	Insufficient number of nuclei (most likely only normal tissue)		8
Total	48	29	19
Total (%)	100	60.4	39.8

### Global instability expressed as total number of breakpoints per genome varied widely in high-grade tumors

We performed array CGH analysis using the 244 K Agilent platform on whole genome amplified DNA isolated from nuclei sorted according to ploidy status. Agilent’s ADM-2 algorithm was used to identify chromosomal segments of altered copy number in individual subpopulations from all tumors. We used CNV calling parameters (threshold and ADM-2 filtrations) established in our previous work to minimized the false positive CNV calls [9]. There are several mechanisms which could lead to genomic rearrangements and a change in copy number in the human genome. They include processes related to DNA replication and recombination as well as DNA repair [10–12]. Many, although probably not all, intra-chromosomal copy number changes seen on array CGH are associated with DNA breakage. In order to quantify genomic breakage-type instability with high precision, we focused on putative DNA breakage points, rather than on the amplifications or deletions themselves. We identified those breakage points as the edges of segments of DNA copy number gains and losses as well as points of abrupt DNA copy number changes called within larger aberrations (for more detail see the Methods section). The precision of breakpoints assessment is determined by the resolution of an array. The smallest aberrations we could detect with confidence on the 244 K Agilent platform are 100 kb in genomic length [9].

**Figure 2:**
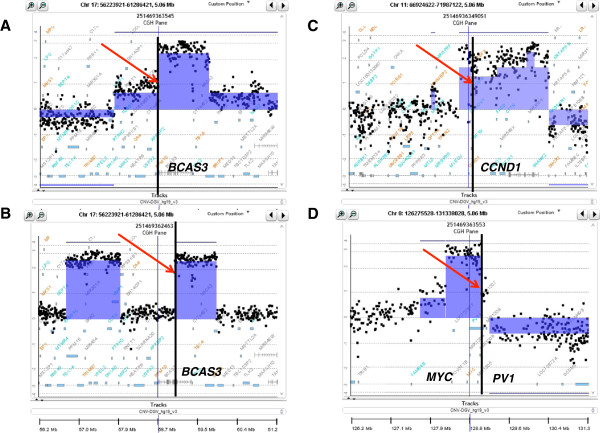
**Examples of putative breakpoints in the areas of frequent genomic rearrangements in breast cancer.** Breakpoints on chromosome 17 **(A and B)**, in two HER2+ genomes, cutting through gene BCAS3, which is often rearranged and fused to other genomic sequences; specimens T111 **(A)** and T333 **(B)**. A breakpoints within the CCND1 gene on chromosome 11 in ER+ tumor; specimen T147 **(C)**. A breakpoints within the PVT1 oncogene at the edge of the amplicon containing the MYC oncogene in a triple negative breast tumor; specimen T199 **(D)**. Each panel shows data points for log 2 ratios of fluorescence between labeled tumor DNA and the differentially labeled normal human reference. Shaded areas identify aberrations called by the ADM-2 algorithm, and the genes are indicated with blue boxes (bottom).

This approach allowed the quantification of genome-wide breakage-related instability in tumors. Examples of putative breakpoints in various tumors are shown in Figure 2. These breakpoints are located at sites of frequent genomic rearrangements and some have previously been shown to be involved in gene fusions (e.g. the BCAS3 gene) [6, 13].

**Figure 3:**
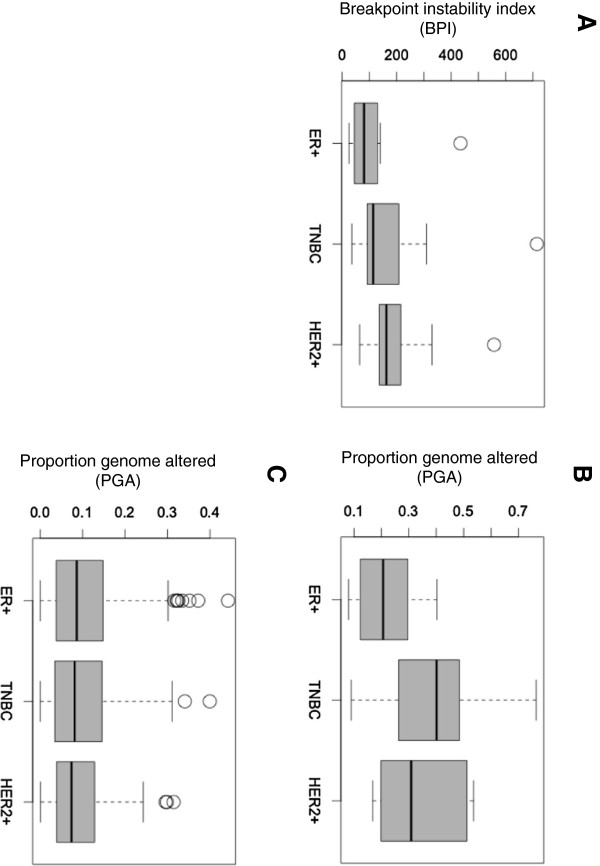
**Chromosome-breakage genomic instability in three clinical subtypes of breast cancer.** Box plot of data representing **A)** Genomic instability calculated for 29 tumors as a total number of putative breakpoints per genome (BPI), **B)** Genomic instability calculated for 29 tumors as proportion genome altered, **C)** Genomic instability in METABRIC cohort [4] expressed as proportion genome altered.

Each tumor was assigned a “breakpoint instability index” (BPI), which is the total number of putative breakpoints per genome. The distribution of BPIs for the three clinical subtypes is shown in Figure 3A. There are no significant differences between median BPI among the 3 clinical subtypes of breast cancer, although there is a trend towards higher BPI in the HER2 and TNBC subtypes. While the majority of tumors in each clinical subtype had BPIs in the range from 25 to 300, there were also a few outliers, that is, tumors which were much more unstable (BPIs > 300). These outliers showed many small aberrations along the genome, and a very large number of breakpoints. The high BPI in these outliers could reflect additional instability mechanisms, or could in part be due to artifacts related to whole genome amplification, previously described by us [9].

The extent of instability expressed as proportion of the genome that is altered (PGA) is shown in Figure 3B. Again, there is no significant difference in PGA between the 3 subtypes. As expected, BPI instability correlates well but not perfectly with the proportion of genome altered (R = 0.418) (Additional file 1: Figure S1). We were able to analyze data on PGA from the Molecular Taxonomy of Breast Cancer International Consortium (METABRIC) [4]. Figure 3C shows the distribution of PGA for 610 unsorted, high risk breast tumors (stage >2) (85 TNBC, 83 HER2+ cancers and 442 ER + tumors) from the METABRIC cohort. Overall these results suggest that all three clinical subtypes of stage 2 breast cancers have similar levels of instability, which varies widely form 0.0001 up to 0.5 of the proportion of the genome that is altered.

### Patterns of instability at the level of individual chromosomes

**Figure 4:**
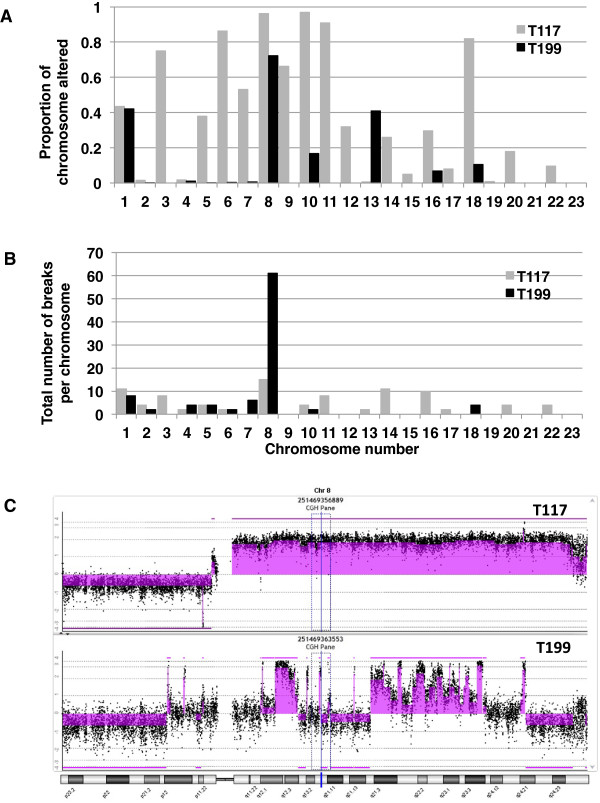
**Chromosome-breakage instability reveals additional complexity of breast cancer genome when shown at the level of individual chromosomes.** The instability at the level of individual chromosomes is shown for two tumors with very similar BPI. If looking at instability as proportion of chromosome altered **(A)** the pattern of instability is very different than that derived from the number of breakpoints per chromosome **(B)**. Views of array CGH data for chromosome 8 in specimen T117 and T199, with aberrations identified with ADM-2 algorithm (shaded areas) showing that Chromosome 8 in tumor T199 is particularly aberrant/broken **(C)**.

As measures of global instability, BPI and PGA provide similar results in our cohort. However, when breakpoint instability is analyzed at the level of individual chromosomes, focusing on breakpoints reveals much additional complexity. This is illustrated in Figure 4. If looking at instability as the proportion of each chromosome that is altered (Figure 4A) the pattern of instability is very different from that derived from the number of breakpoints per chromosome (Figure 4B). It becomes evident that chromosome 8 in tumor T199 is particularly aberrant/broken (Figure 4C).

**Figure 5:**
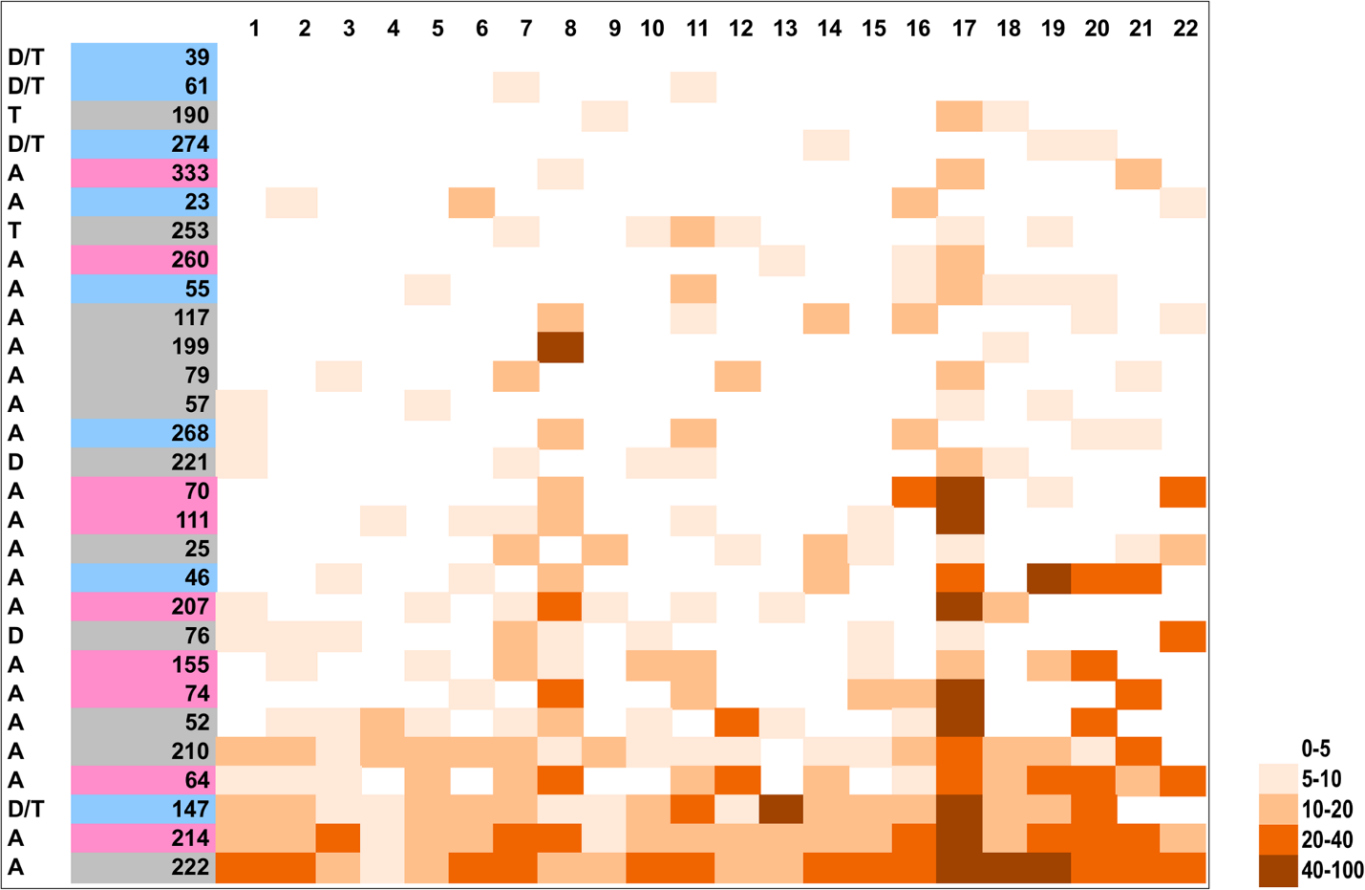
**Densities of breakpoints on individual chromosomes in all tumors.** Heat map showing distribution of breakpoints within genomes. Total number of breakpoints per each chromosome was normalized to the size of the chromosome and it is expressed as numbers of breaks per 100 Mb. Tumors are aligned from the least aberrant (top) to the most aberrant (bottom). Tumor ploidies are indicated on the left: tumors with diploid flow sorting profiles are marked as follows: diploid tumors (D), tetraploid tumor (T), diploid or tetraploid (D/T); tumors with aneuploid flow sorting profiles are marked with A. The color code in the tumor number column corresponds to the clinical subtype: blue = ER+; pink = HER2+; grey = TNBC.

**Figure 6:**
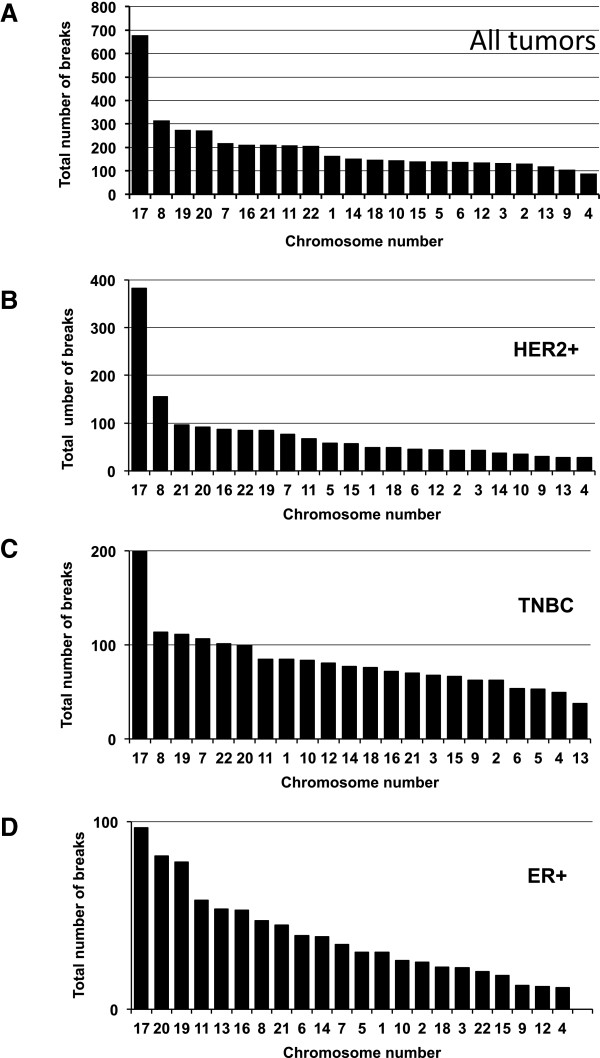
**Distribution of breakpoints between chromosomes in all tumors and in three clinical subtypes.** Total number of breaks per each individual chromosome calculated for all tumors **(A)** and for tumors of each clinical subtypes: HER2+ tumors **(B)** TNBC **(C)** and ER+ tumors **(D)**. Chromosomes are aligned from the most aberrant (left) to the least aberrant (right).

To develop a measure of chromosomal instability, which takes into account the different sizes of the chromosomes, we assessed the density of chromosomal breaks for each chromosome, by normalizing the number of breaks per 100 Mb. The heat map in Figure 5 illustrates the density of chromosomal breaks on individual chromosomes in all tumors. Chromosomal break density was also analyzed separately for each clinical subtype (Additional file 2: Figure S2). Overall, diploid tumors show less breakpoints compared to aneuploid tumors, suggesting that the degree of breakage-related instability is associated with chromosomal-scale instability (ploidy). We then classified the chromosomes from all tumors according to the total number of breaks (Figure 6A). The data plotted for each clinical subtype separately is shown in Figure 6B, C, D. Looking at all tumors together, the highest density of breaks occurred at chromosome 17 followed by chromosome 8 (Figure 5). These two chromosomes also showed the highest total number of breaks in all tumors, followed by chromosome 19, 20, 7 and 16 (Figure 6A). We found that the highest number of breaks occurred on chromosome 17 in all 3 subtypes (Figure 6B, C, D), suggesting a particular proclivity of this chromosome for chromosomal breaks in breast cancers.

The chromosome with the second highest number of breaks was chromosome 8, but only in HER2+ tumors and in TNBCs (Figure 6B and C). On the other hand, ER + tumors showed relatively more breaks on chromosomes 20, 19, 11 and 16 than on chromosome 8 (Figure 6D). Both, chromosomes 11 and 16 have been shown to be aberrant in this clinical subtype [14]. TNBCs also had frequent breaks on chromosomes 19 and 7 (Figure 6C).

### Chromothripsis is present in breast tumors

**Figure 7:**
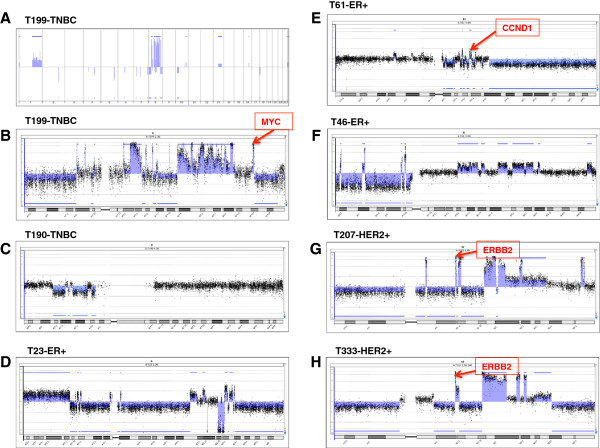
**Patterns of chromothripsis. A)** Genome view of array CGH data form tumor T199 is shown with aberrations identified with ADM-2 algorithm (shaded areas). **B-H)** Views of chromosomes affected by chromothripsis from different tumors; aberrations were identified with ADM-2 algorithm (shaded areas).

The high purity of the flow sorted tumors allowed us to observe chromosomal breaks with great sensitivity. Upon further examination of the patterns of chromosomal instability, it became apparent that a high density of breakpoints on a few individual chromosomes was driving the instability index in many tumors. The most obvious example is the HER2+ subtype, where the tumors with the highest instability indexes displayed many breakpoints on chromosome 17, within and beyond the ERBB2 amplicon. In many tumors, one or two chromosomes contributed predominantly to the high number of breaks. This is especially evident in the less aberrant tumors such as T23, T199 and T190 (Figure 7). We realized that frequently broken chromosomes show break patterns suggestive of chromothripsis as first described by Stephens [6]: the presence of multiple rearrangements confined to one or two chromosomes, sometimes just a small part of one chromosome and the frequent alteration of a limited number (two, sometimes three) of copy number states within affected areas and finally, the loss of heterozygosity (LOH), which, in our case, could not be evaluated with the array CGH platform [5]. Figure 7 and Additional file 3: Figure S3 show examples of those patterns in the genomes from all clinical subtypes, and the data is summarized in Table 2. The most affected chromosomes were again chromosome 17 (55.6% of HER2+ tumors) and chromosome 8 (33.3% of HER2+ tumors), and in many HER2+ tumors the phenomena occurred in tandem (33.3%), affecting both chromosomes 8 and 17. ER + and TN breast cancers did not show chromothripsis on chromosome 17, and 50% of ER + tumors showed chromothripsis on one or two chromosomes with chromosome 11 the most affected. The frequency of chromothripsis was relatively lower (25%) in TNBCs and usually involved only one chromosome.

**Table 2:** **Chromosomes affected by chromothripsis in all tumors**

Tumor	Instability index (BPI)	Clinical type	Chr. 6	Chr. 7	Chr. 8	Chr. 9	Chr. 11	Chr. 17	Chr. 19	Chr. 21	Total per tumor
T 61	30	ER+		x			x				2
T 23	69	ER+	x								1
T 46	139	ER+			x				x		2
T 147	434	ER+					x				1
T 190	35	TNBC				x					1
T 199	93	TNBC			x						1
T 79	104	TNBC		x							1
T 333	64	HER2+	x		x			x			3
T 111	141	HER2+			x			x			2
T 207	162	HER2+						x			1
T 74	215	HER2+			x			x		x	3
T 64	329	HER2+						x		x	2
Total number per all tumors			2	2	5	1	2	5	1	2	

### Clinical correlations

**Figure 8:**
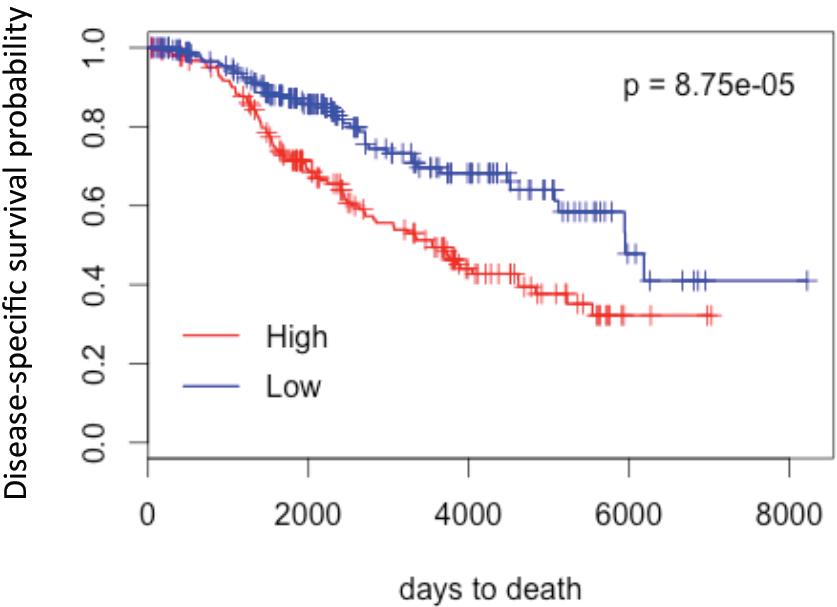
**Genomic instability based on copy number alterations has prognostic value in ER+ breast cancer subtype.** Kaplan-Meier plot of disease-specific survival for 344 patients from METABRIC study [4] (ER+ tumors, stage 2 or higher) divided into high and low instability groups based on the median of proportion genome altered.

Table 3 shows clinical information about the cohort. Tumors of each clinical subtype are classified according to the BPI from the lowest (on the top) to the highest (at the bottom). Disease-free survival and overall survival were measured for all primary tumors. The patients had 61 months of median follow-up. They received adjuvant therapy according to hormone status. Only two of these patients received neoadjuvant chemotherapy and only one received trastuzumab. The number of samples in each subtype was relatively small and did not allow for a statistically significant assessment of correlation between BPI and survival of patients. In ER + tumors, however, there appears to be an inverse correlation between BPI and survival (Additional file 4: Figure S4). Interestingly, three of the 4 ER + tumors with evidence of chromothripsis showed early recurrence (Table 3), which is rather unusual in ER + breast tumors [15]. We verified if genomic instability as measured by proportion genome altered (PGA) could have prognostic value in ER + breast cancers from the METABRIC cohort [4]. Figure 8 shows a Kaplan-Maier plot for disease specific survival for 377 patients with ER + tumors, stage 2 or higher, which were divided into high (n = 188) and low (n = 189) instability groups based on the median of proportion genome altered. These results suggest that the genomic instability based on copy number alterations has prognostic value in the ER + breast cancer subtype.

**Table 3:** **Summary of clinical data for all tumors**

Clinical subtype	Tumor number	Age	T	N	Stage	Grade	ER	PR	HER2+	Recurrence type	DSF (months)	OV (months)	Last follow (months)	BPI	Chromothripsis	Ploidy
ER+	39	72	2	1	2	2	+	+	na	Lung	126		132	25		D/T
	61	61	2	1	2	2	+	+	-	Bone	17		116	30	Yes	D/T
	274	61	3	3	3	na	+	+	-	Liver, bone	35	35		59		D/T
	23	37	4	1	3	1	+	+	na	Bone	57	90		69	Yes	A
	55	52	2	1	2	2	+	+	na	Lung	58	66		91		A
	268	71	2	na	2	2	+	+	-	Liver, bone	45	52		121		A
	46	51	3	2	3	na	+	-	na	Metastasis	23	31		139	Yes	A
	147	32	1	1	2	2	+	+	-	Bone, liver	35	91		434	Yes	D/T
HER2+	333	48	2	0	2	3	-	+	+				18	64	Yes	A
	260	44	3	0	2	2	+	-	+				56	82		A
	70	49	2	1	2	3	-	+	+	Bone, liver	12	35		136		A
	111	44	1	0	1	2	-	-	+	Liver, lung, bone, brain	20	39		141	Yes	A
	207	43	1	0	1	2	-	-	+				43	162	Yes	A
	155	58	1	1	2	3	-	-	+	Brain, bone, liver	26	28		187		A
	74	36	2	1	2	2	+	+	+	Bone	91		110	215	Yes	A
	64	70	2	2	3	3	-	-	+				91	329	Yes	A
	214	38	1	0	1	2	+	+	+				89	557		A
TNBC	190	72	2	0	2	2	-	-	-	Lung, lymph nodes	98		125	35	Yes	T
	253	55	2	0	2	3	-	-	-	Abdomen, lung, bones	71		94	74		T
	117	47	3	2	3	3	-	-	-	Skin, lung	8	26		91		A
	199	44	2	0	2	2	-	-	-	Bone	28	29		93	Yes	A
	79	56	1	1	2	3	-	-	-	Bone, lung	9	29		104	Yes	A
	57	34	1	0	1	3	-	-	-				97	105		A
	221	69	2	2	3	na	-	-	-				61	122		D
	25	45	2	2	3	2	-	-	-				127	150		A
	76	43	1	0	1	3	-	-	-				97	152		D
	52	54	2	0	2	2	-	-	-				85	264		A
	210	45	1	1	2	2	-	-	-	Brain, lung, liver, bone	18	35		309		A
	222	41	2	0	2	3	-	-	-	Brain, bone, liver	17	22		714		A

*Abbreviations* in the table: *T* tumor size category, *N* lymph node status (number of positive nodes), *ER* estrogen receptor status, *PR* progesterone receptor status, *HER2* HER2 receptor status, *DSF* disease free survival (given for the patients who relapsed), *OV* overall (given for the patients who died), *BPI* breakpoints instability index.

## Discussion

Genomic instability is a cardinal feature of solid tumors, including breast cancers, and various forms of instability have been described and measured [16]. The instability as determined by the patterns of DNA amplifications and deletions is prognostic in breast cancer [17–20] although the assessment of global patterns of amplicons and deletions may reduce the complexity of genomic data observed by array CGH. We have described a novel approach for a global analysis of genomic rearrangements in cancer cells, breakpoint analysis, which led us to derive a relatively simple instability index (BPI) from array CGH data, that encompasses all significant chromosomal breaks at individual chromosomes within cancer genome. BPI instability is correlated with commonly used measure of instability, the proportion of genome altered and it may have a prognostic value as well. Most importantly however, when breakpoint instability is visualized at the level of individual chromosomes, it reveals additional complexity.

Using this breakpoints analysis approach, we confirmed the variability in degrees of genomic instability within each breast tumor subtype. Breakpoints inferred from array CGH data were previously used to assess genomic instability in combination with other measures of instability such as a fraction of the genome altered [17]. However, DNA analysis of non flow-sorted specimens underestimates the number and longitudinal complexity of breaks detected by array CGH. Even our approach most likely underestimates the real number of breaks in the genome, since every point of change in copy number may signal many rearrangements and breaks [6] and because of the limited coverage of the array technology we used and the inability to detect balanced translocations. With 244 K Agilent array we are detecting breakpoint regions of about 10 kb in size rather then specific breakpoints. Undoubtedly, we are missing many details, but what we see is sufficient to identify the genomic signature of chromothripsis. Chromothripsis is a complex event and what we are able to detect with array CGH is a snapshot of a final genomic configuration of DNA segments superimposed on the haploid genome, which reflects many breakage events [21]. Thus we should consider the number of breaks described in this study as an estimation. The spectrum of putative breakpoints was very broad for all three clinical subtypes. Moreover, even though most of the tumors in this study were from high-risk patients, some tumors showed only a few breaks and aberrations. These relatively genomically stable tumors were either ER + or, surprisingly, TNBCs.

Many breast tumors in our cohort had numerous rearrangements on one or two chromosomes, while showing fewer aberrations elsewhere. The patterns of instability on these chromosomes were suggestive of chromothripsis. This is the first study reporting patterns of chromothripsis in several breast tumors. Although chromothripsis was initially inferred from sequencing data, it could also be identified with SNP arrays or array CGH by finding regions with altered copy-number with multiple breakpoints localized on one chromosome. Similar patterns of copy-number alternations were associated with chromothripsis in several other studies including the original work by Stephens [6, 22–25]. Interestingly, Hicks observed what they described as “firestorms”, defined as clusters of “a pattern of interdigitated amplifications and LOH” [18]. They also proposed that these events occurred locally and often involve chromosomes 8 and 17. We found that 12 out of 29 (41.4%) high-risk tumors show patterns suggestive of chromothripsis on one or more chromosomes, higher than previous reports in other cancer types [6]. Chromothripsis might be associated with more aggressive tumors [23, 25, 26], which could correspond to our “high risk” tumors. The other reasons for such a high prevalence of chromothripsis in our study could be the fact that our tumor samples were enriched for cancer cells by flow sorting of nuclei, perhaps enabling patterns of altering lower amplitude copy-number changes not to be obscured by normal DNA. Our study is the first to document the prevalence of chromothripsis in a breast cancer cohort, but its prevalence in larger cohort needs to be further studied. The prevalence of chromothripsis in large cohorts could be examined in publicly available array-based copy number data. However, the task is not trivial considering the use of various platforms and different analytical algorithms, as well as a lack of consistency in defining the criteria of chromothripsis [21, 27]. The bioinformatics tools required to identify chromosomes with chromothripsis need to be developed. Focusing on the frequency and patterns of putative breaks on individual chromosomes might be a good start.

Surprisingly, chromosome 17 shows the highest density of breaks in all clinical subtypes. This might be related to the unique structural features of this chromosome, such as the relative abundance of segmental duplications and interspersed repetitive elements, relatively short telomeres, as well as its high gene content and the presence of dosage sensitive genes [28, 29]. In a large evolutionary scale the rearrangements on this chromosome are responsible for human diversity as well as for many hereditary disorders [29]. Chromosome 19 was also one of the most aberrant; similar to chromosome 17, it is very gene dense (in fact, it is the most gene dense chromosome) and contains a high density of repeated sequences [30]. This suggests that genomic instability might not be equal for all chromosomes and that the contribution of individual chromosomes to the development of some tumors may be determined at least in part by their structural features. The second most aberrant chromosome in HER2+ and TNBC tumors (but not in ER + tumors) was chromosome 8. It has been demonstrated that aberrations on chromosome 8 correlate with p53 mutation status and overall survival in breast tumors [31]. ER + tumors showed a relatively high density of breaks on chromosome 11 and chromosome 16, both of which were shown to carry recurrent aberrations [14]. Although our small sample size precluded definitive assessment of the effect of BPI on survival, we show in the large METABRIC cohort that genomic instability derived from copy number analysis may be prognostic in ER + breast cancers. This result is with agreement with previous studies, which showed a significant association of the extent of chromosomal instability with prognosis in ER + but not ER- tumors [30, 32, 33].

## Conclusion

In summary, we used a novel approach to analyze the data obtained with flow-sorted tumor specimens using array CGH, which provides a new insight into the genomic complexity of breast cancer. We found that patterns of genomic instability are different in each clinical subtype, and that chromothripsis contributes to the instability in many high-risk breast tumors. Chromosome 17 was the most “broken” chromosome in all clinical subtypes. These findings suggest that genomic instability dependent on chromosomal breakage events is not stochastic, but occurs in a stepwise fashion, targeting some chromosomes clearly more than others. Our study supports the value of “de-contaminating” tumor samples from stromal cells as a way to increase the sensitivity of genomic analysis, revealing novel insights into the genomic instability patterns underlying breast tumorigenesis.

## Methods

### Patients and specimens

Forty eight primary breast tumors were selected from a large tumor bank of breast tumors obtained from patients undergoing surgery for breast cancer at the Centre Hospitalier de l’Université de Montréal (CHUM) from September 2001 to June 2003. These tumors were selected on the basis of stage 2 or 3 at presentation and/or age < 50 and a balance between the 3 clinical subtypes. We obtained data on clinical follow up until 2012. Patients signed informed consent for tissue banking, and this particular study received approval from the ethics committee of the Jewish General Hospital and the Translational Genomics Research Institute, where the experiments were performed. Samples were flash frozen within an hour after surgery and stored in a -150°C freezer until use.

This study also makes use of data generated by Molecular Taxonomy of Breast Cancer International Consortium (METABRIC). Cancer Research UK and the British Columbia Cancer Agency Branch provided funding for that project [4].

### Flow sorting of nuclei

Nuclei were extracted from tissues and sorted according to a published protocol [8]. Briefly, tumors were minced in the presence of extraction buffer (10 mM Tris pH 7.4, 146 mM NaCl, 2 mM CaCl_2_, 22 mM MgCl_2_, BSA 0.005% and Igepal CA-630 0.1%) containing DAPI (final concentration 10 μg/ml). The suspension was passed through a 20 G needle to further disaggregate nuclei and was filtered through a 40-μm mesh. Nuclei were sorted according to DAPI intensity using an In flux cytometer (Becton-Dickinson) with UV excitation and DAPI emission collected at > 450 nm. DNA content and cell cycle were analyzed using the software program MultiCycle (Phoenix Flow Systems). DNA from nuclei was isolated using QIAmp DNA MicroKit (Qiagen #56304) according to the manufacturer’s directions for genomic DNA isolation from tissues.

### Whole genome amplification

We used 100 ng of both tumor and reference DNA for each analysis. Whole genomic DNA was amplified using GenomiPhi V2 DNA Amplification Kit (GE Healthcare UK Limited, Buckinghamshire, UK), which uses random primers to target the entire DNA template and φ29 DNA polymerase. The final yield of labeled genomic DNA for hybridization on the array was 7–10 μg of both tumor and reference DNA.

### Array CGH

Copy number alterations in tumor DNA were determined relative to the sex-matched normal human DNA (Promega, Madison, WI) and were identified by array CGH analysis using microarray slides, which contain 244 000 (244 K) oligonucleotide probes (Agilent Technologies, Santa Clara, CA, USA). For sample preparation and hybridization we followed the manufacturer’s protocol. Briefly, amplified DNA was labeled by random priming using either Cy5-dUTP or Cy3-dUTP. Following purification with Microcon Centrifugation Filters, Ultracel YM-30 (Millipore, Billerica, Ma, USA). Probes were denatured and pre-annealed with 50 μg of human Cot-1 DNA (Invitrogen, Burlington, Ontario, Canada). Hybridization was performed at 65°C for 40 h with constant rotation. After hybridization, slides were washed according to the manufacturer’s instructions and scanned immediately with a DNA Microarray Scanner (Agilent Technologies). Data were extracted from scanned images using Feature Extraction software, version 10.7.3.1 (Agilent). The text files were then imported for analysis into Genomic Workbench, standard edition 6.5.0.58 (Agilent).

### Aberration detection

We used the Aberration Detection Method 2 (ADM-2) algorithm to identify DNA copy number aberrations. Agilent’s ADM-2 algorithm identifies all aberrant intervals in a given sample with consistently high or low log ratios based on the statistical score. It then samples adjacent probes to arrive at an estimation of the true range of the aberrant segment. The statistical score represents the deviation of the average of the log ratios from the expected value of zero, in units of standard deviation. The algorithm searches for intervals in which a statistical score based on the average quality weighted log ratio of the sample and reference channels exceeds a user specified threshold. To minimize the false positives we used array CGH platform and CNV calling parameters (threshold and filtration) previously elaborated and reported by us [9]. This paper compared CNVs detected by array CGH performed on whole genome amplified DNA with those detected when array CGH was performed on non- amplified DNA from MCF-7 breast cancer cells. Although a threshold of 6 is recommended in the instruction manual, we used a conservative threshold of 10 because visual inspection of the array plots led to the rejection of several aberrations called using the lower threshold. We applied a filtering option of minimum of 5 probes in region and minimum absolute average log_2_ ratio > 0.3 to define aberrations. UCSC human genome assembly hg19 was used as a reference.

Breakpoints were then identified as locations where a change in copy number occurred as determined by ADM-2. All putative breaks were filtered according to a difference in the mean log_2_ ratio > 0.5 between two consecutive intervals. This filtration decreased the number of breaks by 50% (±12%). When these criteria were used, the only breakpoints found in normal diploid fractions were associated with known germ line copy number variations (CNV) (Figure 1). Thus our normal diploid fractions served as a “negative control” to understand the false positive rate. The fraction of the genome altered was calculated by summing the lengths of all regions with either amplification or deletion, as determined by ADM-2.

## Electronic supplementary material

Additional file 1: Figure S1: BIP instability is correlated with the proportion of genome altered. The genomic instability derived from array CGH data and expressed as BPI is plotted for 29 breast tumors in function of proportion genome altered. (PDF 40 KB)

Additional file 2: Figure S2: Densities of breakpoints on individual chromosomes in three clinical subtypes. A-C) Heat maps showing distribution of breakpoints within genomes for three clinical subtypes separately. Total number of breakpoints per each chromosome was normalized to the size of the chromosome and it is expressed as numbers of breaks per 100 Mb. In each panel tumors are aligned from the least aberrant (top) to the most aberrant (bottom). Tumor ploidies are indicated on the left: tumors with diploid flow sorting profiles are marked as follows: diploid tumors (D), tetraploid tumor (T), diploid or tetraploid (D/T); tumors with aneuploid flow sorting profile are marked with A. The color code in the tumor number column corresponds to the clinical subtype: blue = ER+; pink = HER2+; grey = TNBC. (PDF 282 KB)

Additional file 3: Figure S3: Patterns of chromothripsis. A-H) Views of chromosomes affected by chromothripsis from different tumors; aberrations were identified with ADM-2 algorithm (shaded areas). (ZIP 364 KB)

Additional file 4: Figure S4: Patients overall survival plotted in function of BPI. Overall survival plotted in function of BPI for patients with ER+ tumors **(A)** TNBC tumors **(B)** and HER2+ **(C)**. The plots represent the data from Table 3. Empty symbols stand for the surviving patients. (ZIP 90 KB)

## Abbreviations

ADM-2: Aberration detection method 2
Array CGH: Array comparative genomic hybridization
BPI: Breakpoint instability index
CNV: Copy number variations
ER: Estrogen receptor
ER+: Estrogen receptor positive
HER2: Human epidermal growth factor receptor 2
HER2+: Human epidermal growth factor receptor 2 overexpressing
IHC: Immunohistochemistry
LOH: Loss of heterozygosity
PGA: proportion of genome altered
PR: Progesterone receptor
SNP: Single nucleotide polymorphism
TN: Triple negative
TNBC: Triple negative breast cancers
244 K array: Array CGH containing 244 000 oligonucleotide probes
UCSC: University of California, Santa Cruz.
