# Supplementary figures and images for: Chromosome-breakage genomic instability and chromothripsis in breast cancer
Source: BMC Genomics. 2014 Jul 9;15(1):579. doi: 10.1186/1471-2164-15-579 (PMC4227294; doi:10.1186/1471-2164-15-579)

Figure S1. BIP instability is correlated with the proportion of genome altered

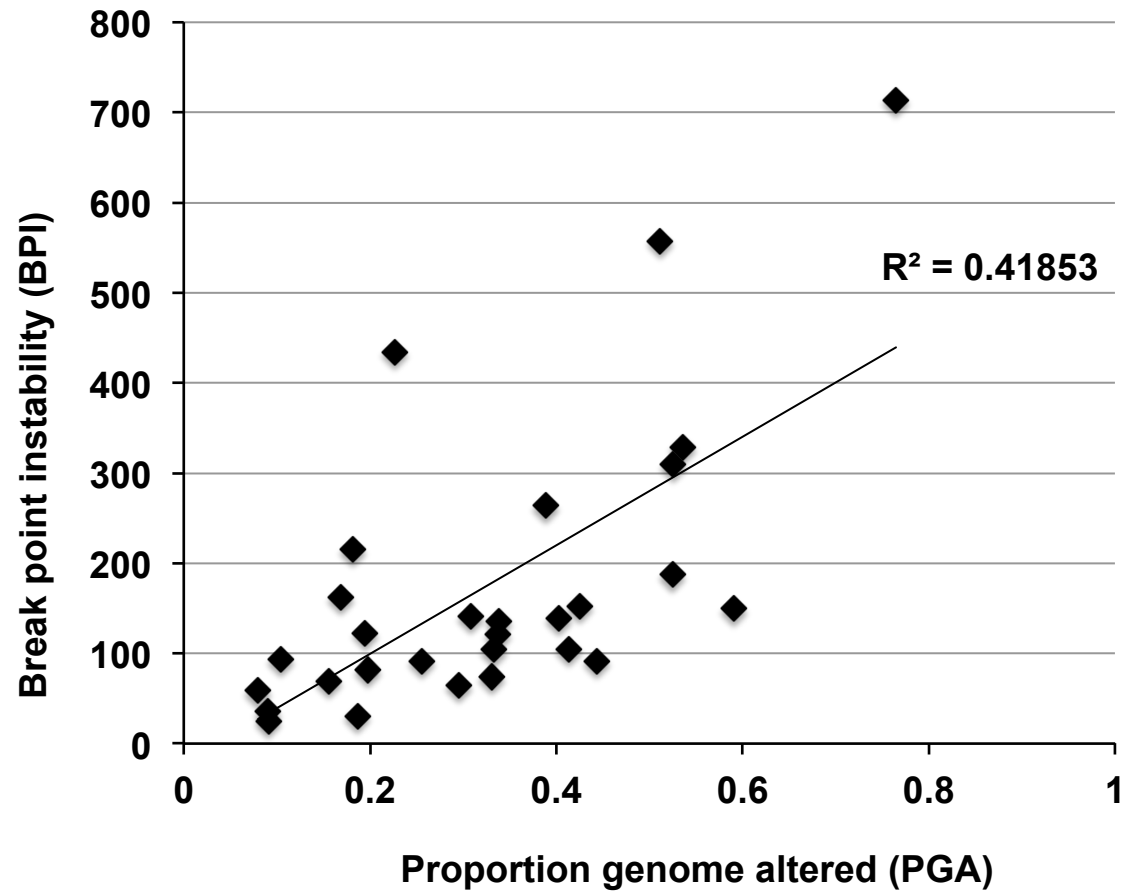

Supplement: Supplementary file 1 — Additional file 1: Figure S1: BIP instability is correlated with the proportion of genome altered. The genomic instability derived from array CGH data and expressed as BPI is plotted for 29 breast tumors in function of proportion genome altered. (PDF 40 KB) [file 12864_2014_7063_MOESM1_ESM.pdf]

Figure S2. Densities of break-points on individual chromosomes in three clinical subtypes.

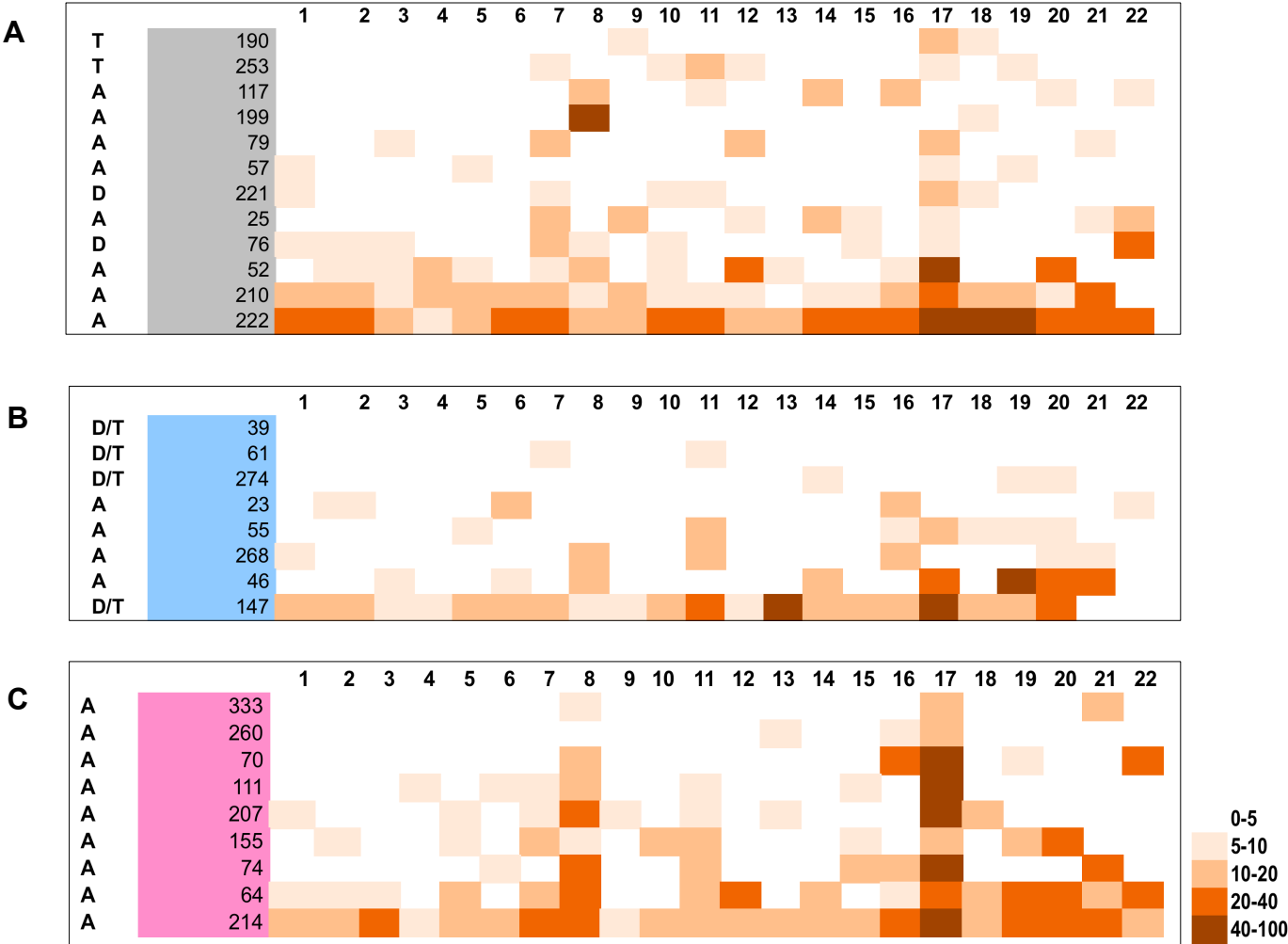

Supplement: Supplementary file 2 — Additional file 2: Figure S2: Densities of breakpoints on individual chromosomes in three clinical subtypes. A-C) Heat maps showing distribution of breakpoints within genomes for three clinical subtypes separately. Total number of breakpoints per each chromosome was normalized to the size of the chromosome and it is expressed as numbers of breaks per 100 Mb. In each panel tumors are aligned from the least aberrant (top) to the most aberrant (bottom). Tumor ploidies are indicated on the left: tumors with diploid flow sorting profiles are marked as follows: diploid tumors (D), tetraploid tumor (T), diploid or tetraploid (D/T); tumors with aneuploid flow sorting profile are marked with A. The color code in the tumor number column corresponds to the clinical subtype: blue = ER+; pink = HER2+; grey = TNBC. (PDF 282 KB) [file 12864_2014_7063_MOESM2_ESM.pdf]

**Figure S3. Patterns of chromothripsis**

**T61-ER+**

**A**

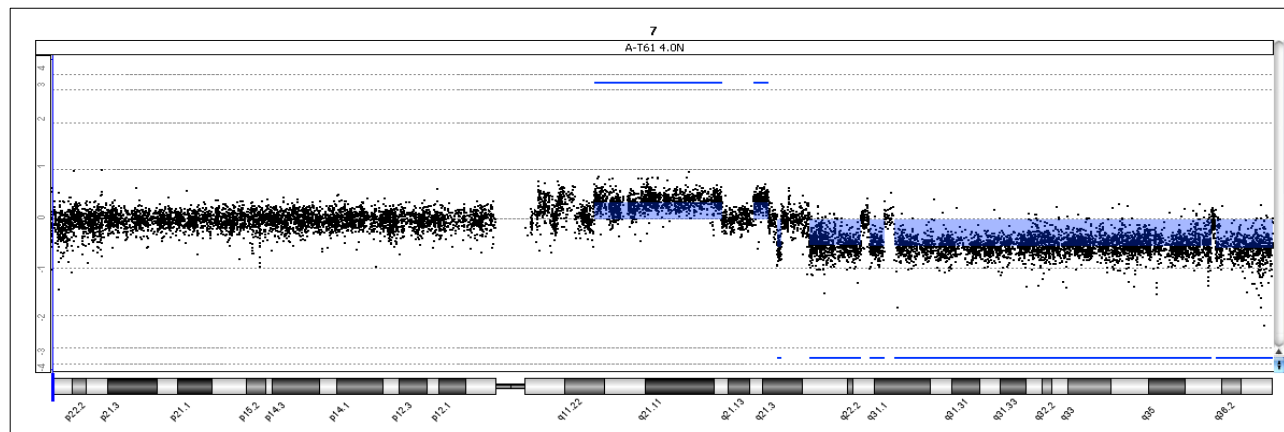

**T46-ER+**

**B**

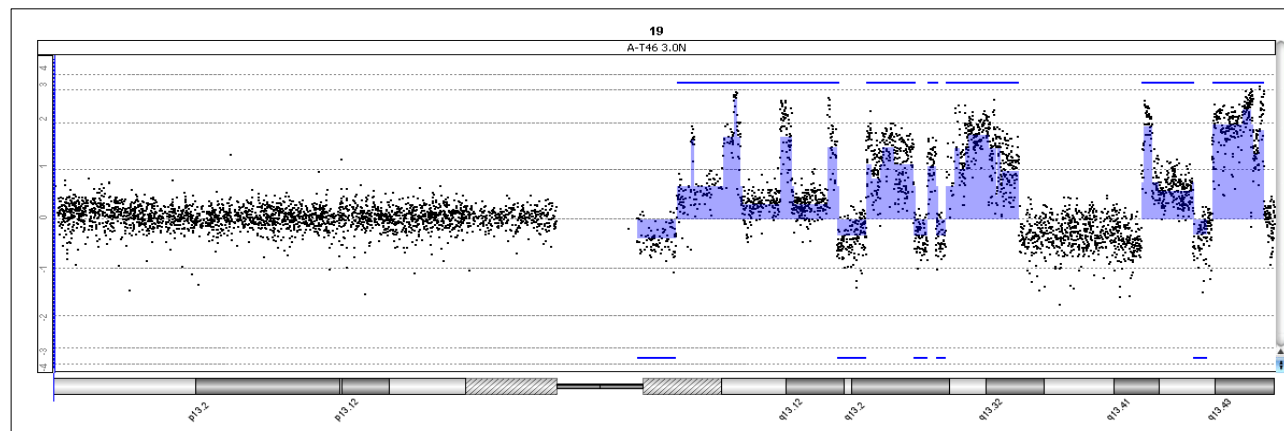

Supplement: Supplementary file 3 — Additional file 3: Figure S3: Patterns of chromothripsis. A-H) Views of chromosomes affected by chromothripsis from different tumors; aberrations were identified with ADM-2 algorithm (shaded areas). (ZIP 364 KB) [file 12864_2014_7063_MOESM3_ESM.zip › 1325747889120650_add3/1325747889120650_add3A and 3B.pdf]

## T79-TNBC

C

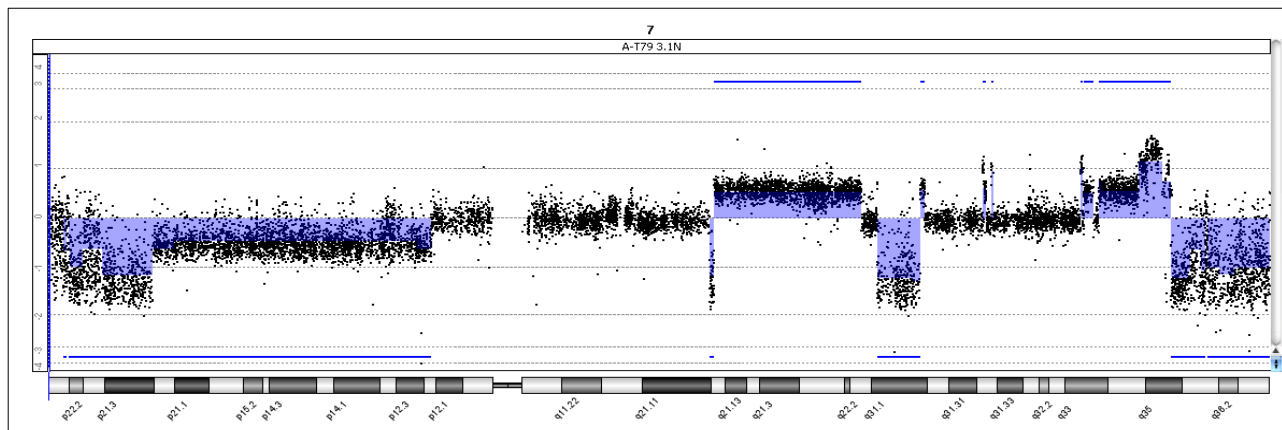

## T111-HER2+

D

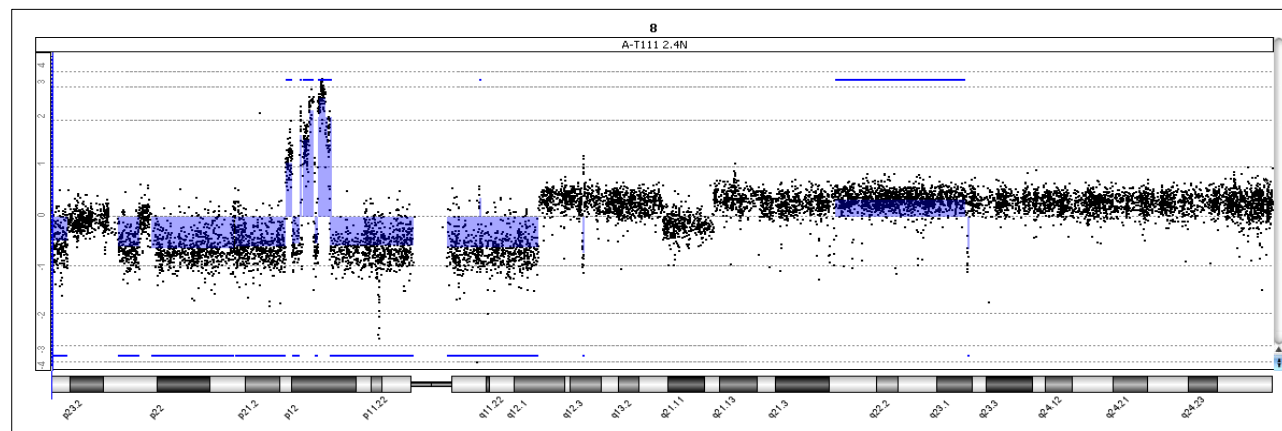

Supplement: Supplementary file 3 — Additional file 3: Figure S3: Patterns of chromothripsis. A-H) Views of chromosomes affected by chromothripsis from different tumors; aberrations were identified with ADM-2 algorithm (shaded areas). (ZIP 364 KB) [file 12864_2014_7063_MOESM3_ESM.zip › 1325747889120650_add3/1325747889120650_add3C and 3D.pdf]

# T333-HER2+

E

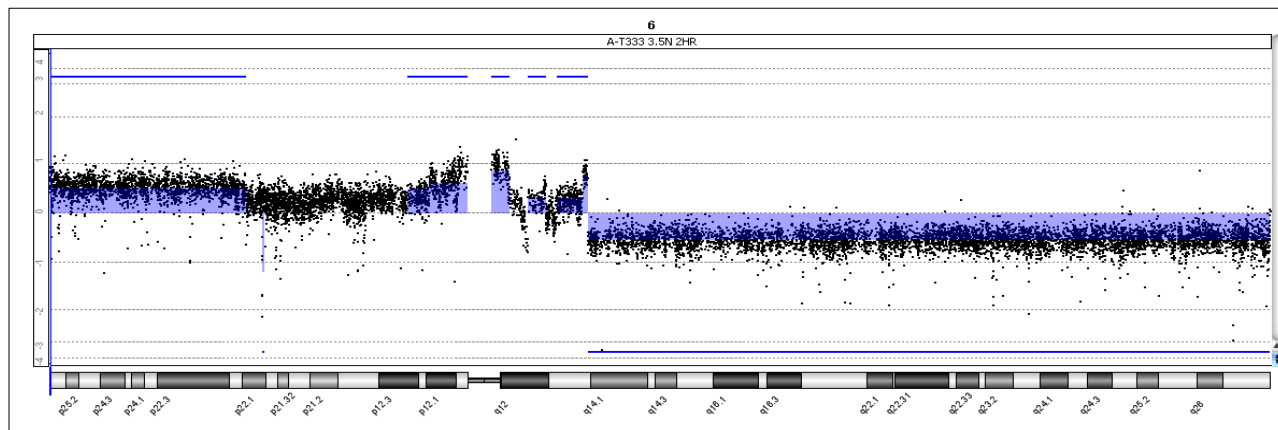

# T333-HER2+

F

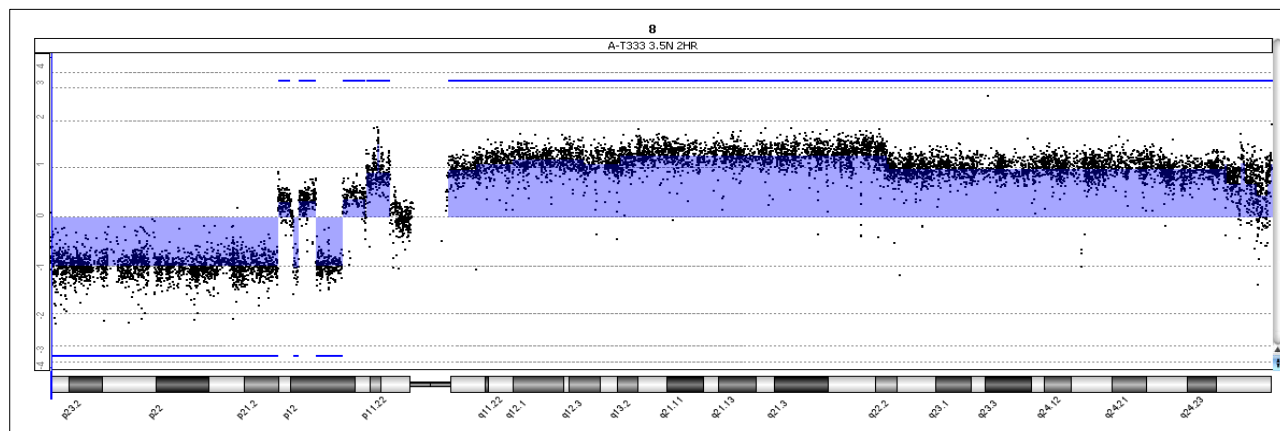

Supplement: Supplementary file 3 — Additional file 3: Figure S3: Patterns of chromothripsis. A-H) Views of chromosomes affected by chromothripsis from different tumors; aberrations were identified with ADM-2 algorithm (shaded areas). (ZIP 364 KB) [file 12864_2014_7063_MOESM3_ESM.zip › 1325747889120650_add3/1325747889120650_add3E and 3F.pdf]

# T111-HER2+

G

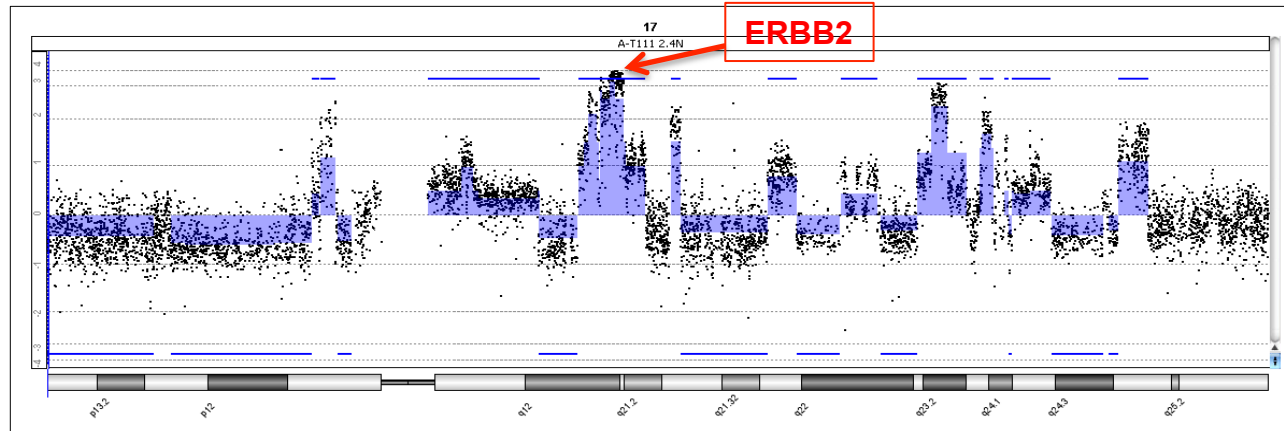

# T74-HER2+

H

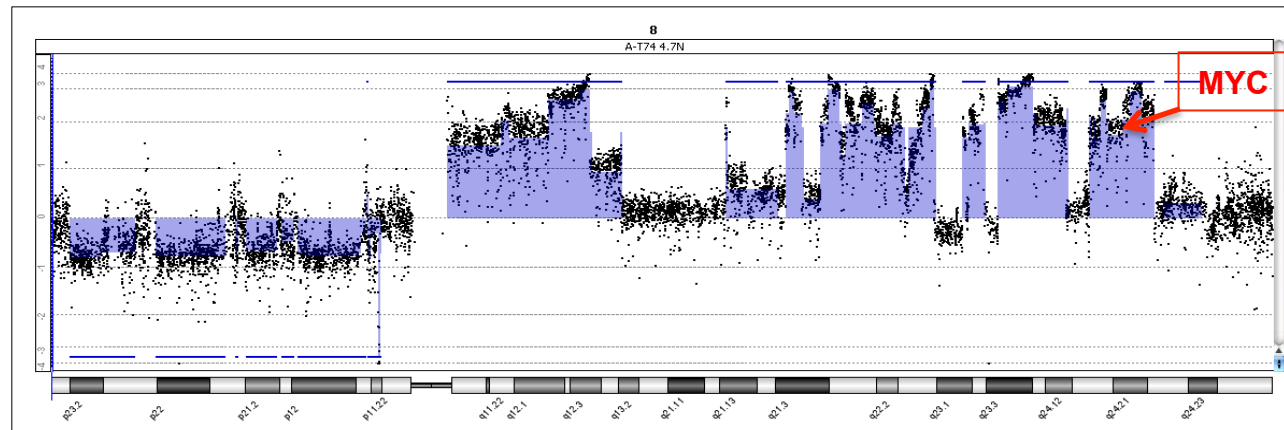

Supplement: Supplementary file 3 — Additional file 3: Figure S3: Patterns of chromothripsis. A-H) Views of chromosomes affected by chromothripsis from different tumors; aberrations were identified with ADM-2 algorithm (shaded areas). (ZIP 364 KB) [file 12864_2014_7063_MOESM3_ESM.zip › 1325747889120650_add3/1325747889120650_add3G and 3H.pdf]

Figure S4. Patients overall survival plotted in function of BPI.

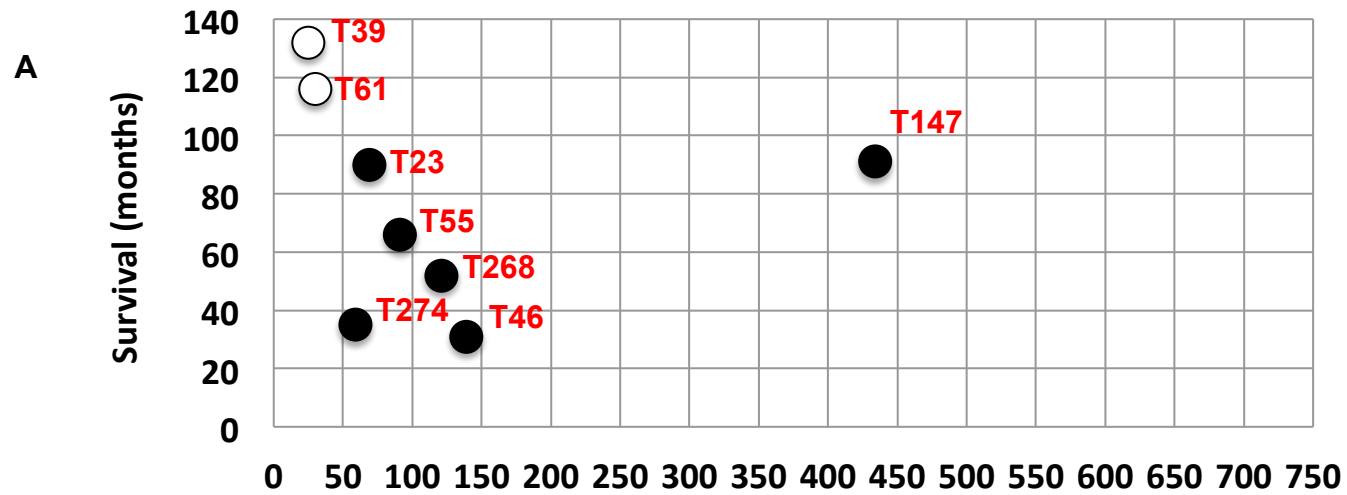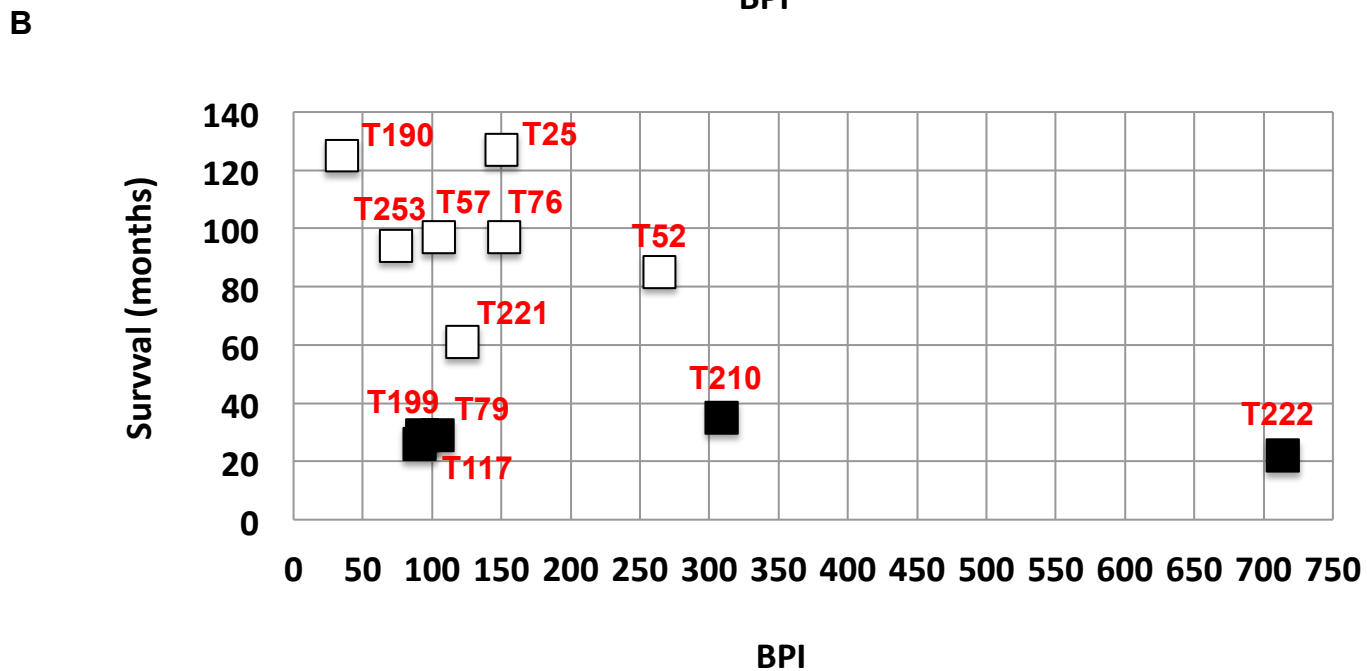

Supplement: Supplementary file 4 — Additional file 4: Figure S4: Patients overall survival plotted in function of BPI. Overall survival plotted in function of BPI for patients with ER+ tumors (A) TNBC tumors (B) and HER2+ (C). The plots represent the data from Table 3. Empty symbols stand for the surviving patients. (ZIP 90 KB) [file 12864_2014_7063_MOESM4_ESM.zip › 1325747889120650_add4/1325747889120650_add4A and 4B.pdf]

C

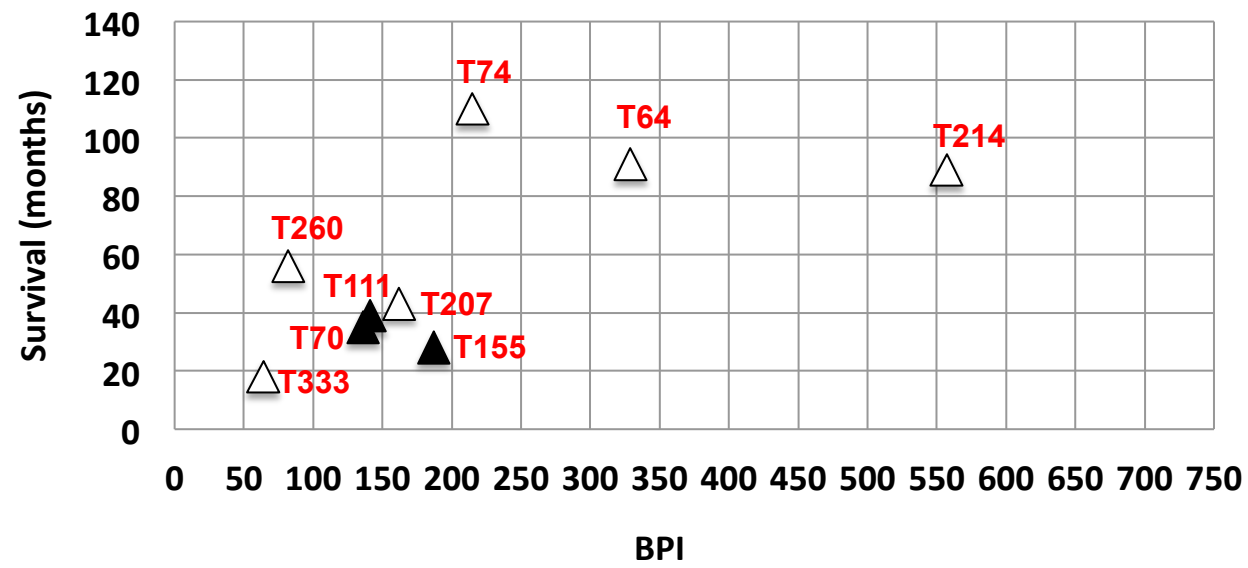

Supplement: Supplementary file 4 — Additional file 4: Figure S4: Patients overall survival plotted in function of BPI. Overall survival plotted in function of BPI for patients with ER+ tumors (A) TNBC tumors (B) and HER2+ (C). The plots represent the data from Table 3. Empty symbols stand for the surviving patients. (ZIP 90 KB) [file 12864_2014_7063_MOESM4_ESM.zip › 1325747889120650_add4/1325747889120650_add4C.pdf]
